# The oncoplastic breast surgery with pedicled omental flap harvested by laparoscopy: initial experiences from China

**DOI:** 10.1186/s12957-015-0514-9

**Published:** 2015-03-07

**Authors:** Dandan Guan, Hui Lin, Zhenye Lv, Ying Xin, Kexin Meng, Xiangyang Song

**Affiliations:** Department of Breast and Thyroid Surgery, Zhejiang Provincial People’s Hospital, 158 Shang Tang Road, Hangzhou, 310014 Zhejiang Province China; Department of General Surgery, Sir Run Run Shaw Hospital, School of Medicine, Zhejiang University, 3 East Qing Chun Road, Hangzhou, 310016 Zhejiang Province China

**Keywords:** Breast cancer, Breast-conserving surgery, Oncoplastic breast surgery, Omental flap, Laparoscopy

## Abstract

**Background:**

A new technique of oncoplastic breast surgery (OBS) using laparoscopically harvested pedicled omental flap has been developed in the past 10 years. This study aimed to evaluate the feasibility of this technique.

**Methods:**

Twenty-five patients underwent OBS using laparoscopically harvested omental flap. Operative time, blood loss, complications, recurrence, and cosmetic outcomes were prospectively analyzed.

**Results:**

Between June 2010 and March 2014, 25 patients were recruited in our study. The surgery was performed successfully in 24 patients. All these patients recovered uneventfully after the surgery. Mean operative time was 310 min, ranging from 205 to 410 min. Mean blood loss was 70 ml, ranging from 20 to 150 ml. Patients were followed up for 32 months on average, ranging from 6 to 51 months. Four patients complained of mild epigastric discomfort. One patient had local recurrence and distant bone and liver metastasis and died 11 months after the surgery. One patient was diagnosed with metastases in the lung, bone, and liver without local recurrence 2 years after surgery. The cosmetic satisfaction rate was 91.7% and 95.8% by surgeon and patients, respectively.

**Conclusion:**

OBS with laparoscopically harvested omental flap might be a feasible technique with a good cosmetic outcome.

## Background

Breast-conserving surgery (BCS) could provide better esthetic results when compared to mastectomy. It has become one of the mainstream surgery for early breast cancer [1,2]. However, BCS is still not the first option for many patients in China. A nationwide survey in China showed that mastectomy continues to account for 88.8% of surgery for primary breast cancer [3]. Most Chinese women have smaller-size breasts with larger tumors found at diagnosis. As a result, a tumor as small as 3 cm may require mastectomy or breast reconstruction. Approximately 10% to 30% of patients submitted to BCS may experience breast deformity or defect [4]. Therefore, a solution to this cosmetic problem is necessary.

Since the early 21st century, European scholars reported breast reconstruction with laparoscopically obtained omental flap successively [5,6]. A few years later, Zaha from Japan reported their experiences on breast reconstruction with laparascopically harvested omental flap after BCS and demonstrated the safety and feasibility of this procedure [7-9].

On that basis, we supposed that the technique of immediate oncoplastic breast surgery (OBS) using laparoscopically harvested omental flap might be suitable for Chinese women with small breasts who would like to accept BCS. We explored this technique in breast cancer patients in China.

## Methods

Between June 2010 and March 2014, 25 patients underwent immediate OBS using laparoscopically harvested omental flap after BCS. All portions of the operation were performed by the same surgical team. The characteristics of the patients were listed in Table 1. All the patients were chosen for BCS with the expectation of postoperative deformity or defect (the estimated resection volume over 25%). The patients who had contraindications of BCS or history of intra-abdominal malignancy, abdominal inflammation, or upper abdominal surgery were excluded from the study. The study was approved by the Research Ethics Committee of our institution. Written informed consent for this study was obtained from the patients for the publication of this report and any accompanying images.

**Table 1:** **Patients’ characteristics**

	***N***
Follow-up time (months)	32 (6 to 51)
Age (years)	43 (30 to 56)
Tumor size (cm)	2.5 (1.5 to 5)
Tumor location	
Outer upper quadrant	14
Outer lower quadrant	3
Inner upper quadrant	3
Inner lower quadrant	5
Pathological diagnosis	
Invasive ductal carcinoma	22
Intraductal carcinoma *in situ*	2
Benign phyllode tumors	1
Comorbidities	
Hypertension	2
Cholecystolithiasis	1
Syphilis	1
Right nephrolith, renal hamartoma, and urinary tract infection	1
Preoperative chemotherapy	2

Breast ultrasound, mammography, and magnetic resonance imaging were performed in all patients. A pathological result was obtained by core needle biopsy before surgery. Distant metastasis was ruled out by preoperative examinations including chest diagnostic CT, abdominal CT, and bone scan. Gastroscopy was performed in each patient to rule out gastric carcinoma. Preoperative chemotherapy for BCS was permitted. Postoperative adjuvant therapy and follow-up were carried out according to the NCCN breast cancer guidelines. Whole breast irradiation was routinely delivered.

The cosmetic evaluation system proposed by Kroll [10] was adopted including symmetry, shape, ptosis, and scars (Table 2). The cosmetic outcome was classified into four grades according to the four-category Harvard scale [11]. The result was defined as excellent, good, fair, and poor. The surgeon and patients assessed the cosmetic outcome independently. ‘Satisfaction’ would be given when the cosmetic outcome was assessed as ‘excellent’ or ‘good’.

**Table 2:** **Assessment standards for cosmetic results**

**Grade**	**Symmetry**	**Shape**	**Ptosis**	**Scars**
Excellent	Symmetrical	Normal	Nature	No contracture
Good	Slightly asymmetrical	Slight deformation	Slightly unnatural	Slight contracture
Fair	Asymmetrical	Deformity	Unnatural	Contracture
Poor	Very asymmetrical	Severe deformity	Very unnatural	Severe contracture

### Surgical procedures

During the operation, OBS with laparoscopically harvested omental flap would be performed to reconstruct the breast immediately after BCS and sentinel node biopsy (SNB) were accomplished. The surgical management was as follows:

1. Preparations before surgery

The inframammary crest, middle sternal line, tumor location, surgical incision, and tumor resection range were marked in a supine position under general anesthesia before surgery. Surgery then was performed in the supine position with the ipsilateral arm resting at 90° abduction.

2. Evaluation and harvest of the omental flap

A camera port (10-mm 30°) was inserted under the umbilicus, and additional three ports were placed. One 10-mm port and a 5-mm port for the surgeon were inserted from the right abdominal wall. The former was at the level of the umbilicus through the right midclavicular line. One 5-mm port for the assistant was inserted at the opposite position of the 10-mm port. Pneumoperitoneum was maintained at 10 mmHg. Then, laparoscopic inspection of the entire abdominal cavity was performed. The omentum was evaluated for size, thickness, vascular supply, and adhesion.

The omentum was dissected upward and dissected in an avascular plane from the splenic flexure to the hepatic flexure of the colon. The left gastroepiploic artery and vein (GEAV) were carefully identified and dissected from the spleen. The right GEAV was preserved as the pedicle of the omental flap. GEAV branches to the stomach wall along the greater curvature were dissected from the left to right afterwards.

3. BCS

BCS was then performed after the omentum was harvested. Quadrant resection or a wider segment resection with an adequate margin was usually performed to minimize risk of re-excision. The excision margins were evaluated by intraoperative frozen sections to confirm complete resection of the tumor. Once the margins were reported involved or close (<1 mm), a wider excision was carried out immediately and a second frozen section was needed to confirm the negative margin. SNB was performed for cN0 breast cancer patients. If the sentinel lymph nodes (SLNs) were confirmed positive intraoperatively by frozen section, subsequent axillary lymph nodes dissection (ALND, levels I and II) would be carried out. For cN1 breast cancer patients, ALND was performed routinely.

4. Creation of omentum export

For the passage of the omentum, a subcutaneous tunnel was prepared from the xiphoid process toward the defect, starting with a 5-cm-long incision along the medial part of the inframammary crest. The mammary tissue along the tunnel was dissected off the pectoralis muscle to extend the space (except the inner lower quadrant tumors). Removal of mammary tissue was unnecessary. A longitudinal incision 4 to 5 cm long along the linea alba just below the xiphoid process was made to communicate with the abdominal cavity. The harvested omentum was pulled out from the enterocoelia gently through the tunnel and unfolded to assess the volume and intact blood supply.

5. Breast reconstruction

The omental flap was replaced over the pectoralis major muscle to fill the defect space in the breast. The omental flap was shaped to adequately fill the breast defect space, without any twist. The subxiphoid linea alba incision was closed, leaving enough space (2 cm) to accommodate the pedicle tissue in the tunnel. It was not required to fix or suture the omentum in place with breast tissue or muscle to maintain its shape and orientation. Two drains were separately placed in the omental filled area and axilla. If these two areas were integrated, only one drain was placed.

## Results

**Figure 1:**
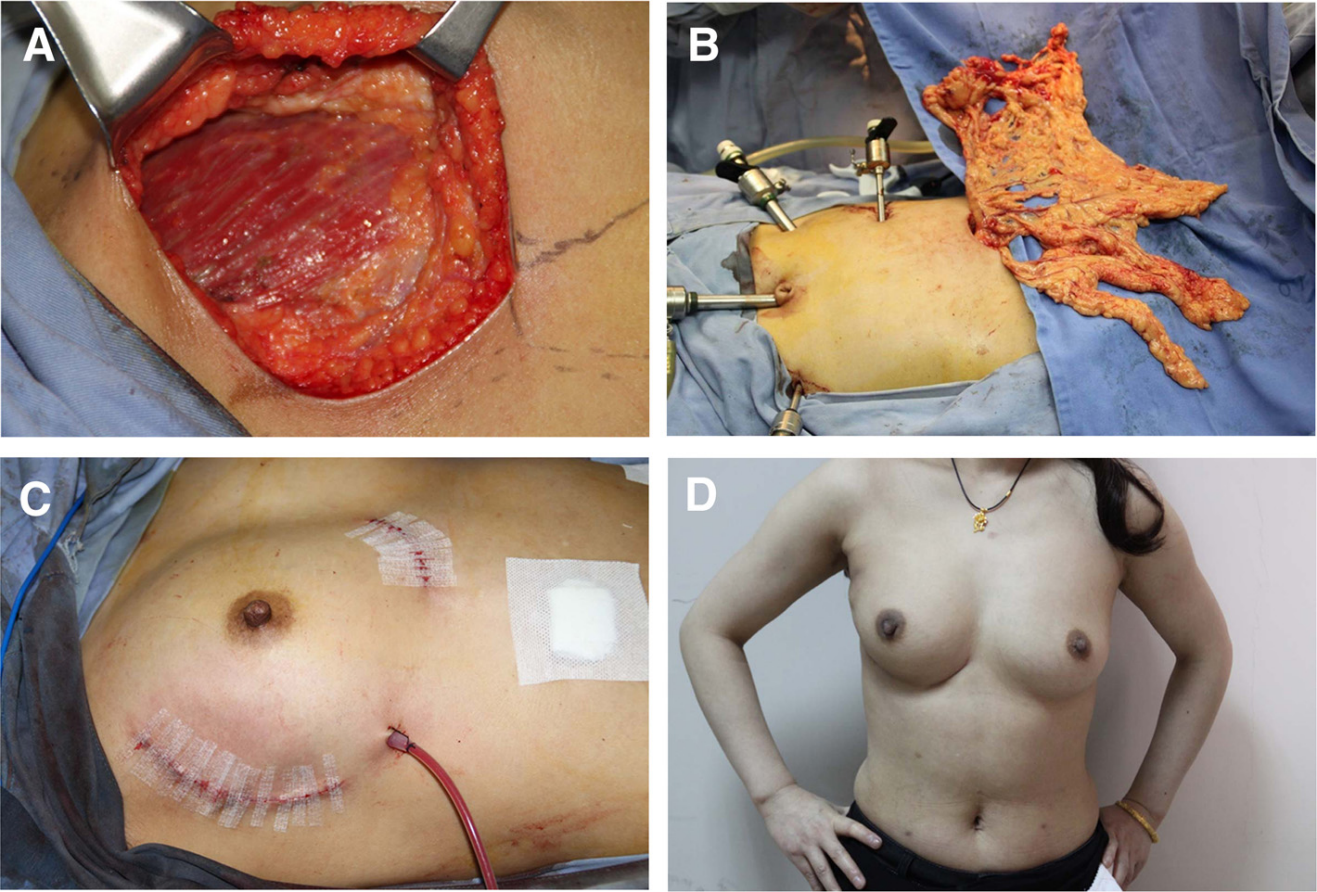
**The omentum fills the defect well. (A)** Upper outer quadrantectomy in the right breast. **(B)** Laparoscopically havested omentum with vessel pedicle. **(C)** Oncoplasty with omentum. **(D)** The outcome at 7 months after surgery, 3 months after radiation.

Between June 2010 and March 2014, 25 patients were enrolled and went through OBS with laparoscopically harvested pedicled omental flap. The surgical outcomes were listed in Table 3. The surgery was performed successfully in 24 patients. The omental flap was easily shaped and fit the defect well (Figure 1). One case was suspended due to severe abdominal adhesions. Three cases had involved or close (<1 mm) surgical margins. Extended resection was then carried out, and the second intraoperative frozen sections now confirmed the negative margin. No case was converted to laparotomy.

**Table 3:** **Surgical outcomes**

	***N***
Total surgery	25
Surgical success	24
Omentum harvest failed	1
Conversion to laparotomy	0
Involved/close margin for the first resection	3
Axilla lymph nodes	
ALND	10
SLNB (+)	4
SLNB (−)	10
Mean operation time	310 (205 to 410) min
Time of omentum harvest	70 (40 to 110) min
Mean blood loss	70 (20 to 150) ml
Blood loss associated with laparoscopy	20 (0 to 70) ml
Hospital stays	9 (7 to 14) days

**Figure 2:**
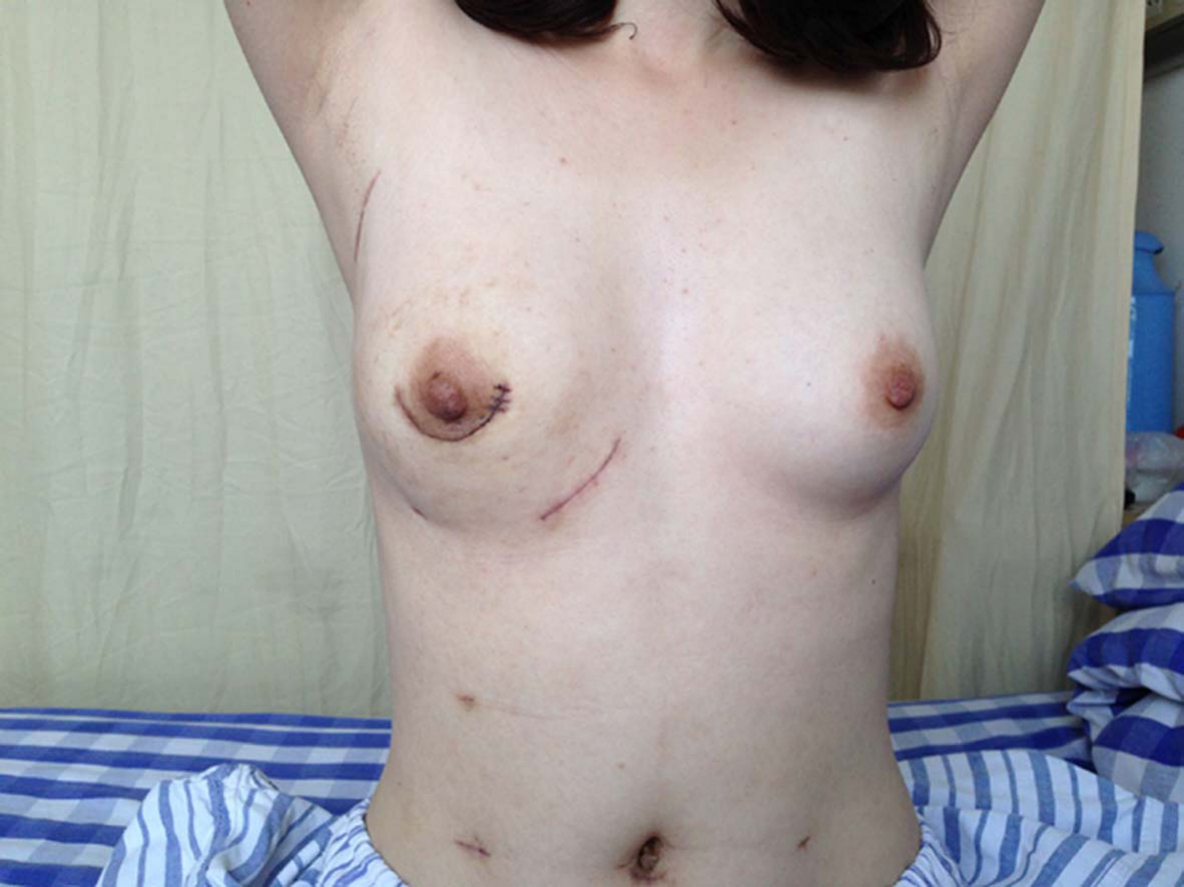
**Minimal donor-site morbidity, postoperative 6 weeks.**

All patients recovered uneventfully after surgery. The donor site was minimally invaded (Figure 2). Drains were removed when the residue volume was less than 30 ml for two successive days. Patients were followed up for 32 (6 to 51) months on average. The complications and oncologic outcomes were showed in Table 4. There were four cases of mild epigastric discomfort, which was relieved spontaneously 2 to 3 weeks later. There were three cases of small fat necrosis nodules with a diameter of 1 to 2 cm in the omental flap without affecting the appearance of the reconstructed breast. No complications such as abdominal hemorrhage, bowel obstruction, bowel perforation, incision hernia, incision infection, and the subcutaneous seroma were found.

**Table 4:** **Complications and oncologic outcomes**

**Complications and oncologic outcomes**	**N**
Epigastric discomfort	4
Necrotic nodules in the omental flap	3
Omentum scald necrosis by accident	1
Complications related with laparoscopy	0
Complications related with breast surgery	0
Distant metastases, no local recurrence	1
Death, distant and local recurrence	1
Off-protocol in postoperative therapy	1

**Figure 3:**
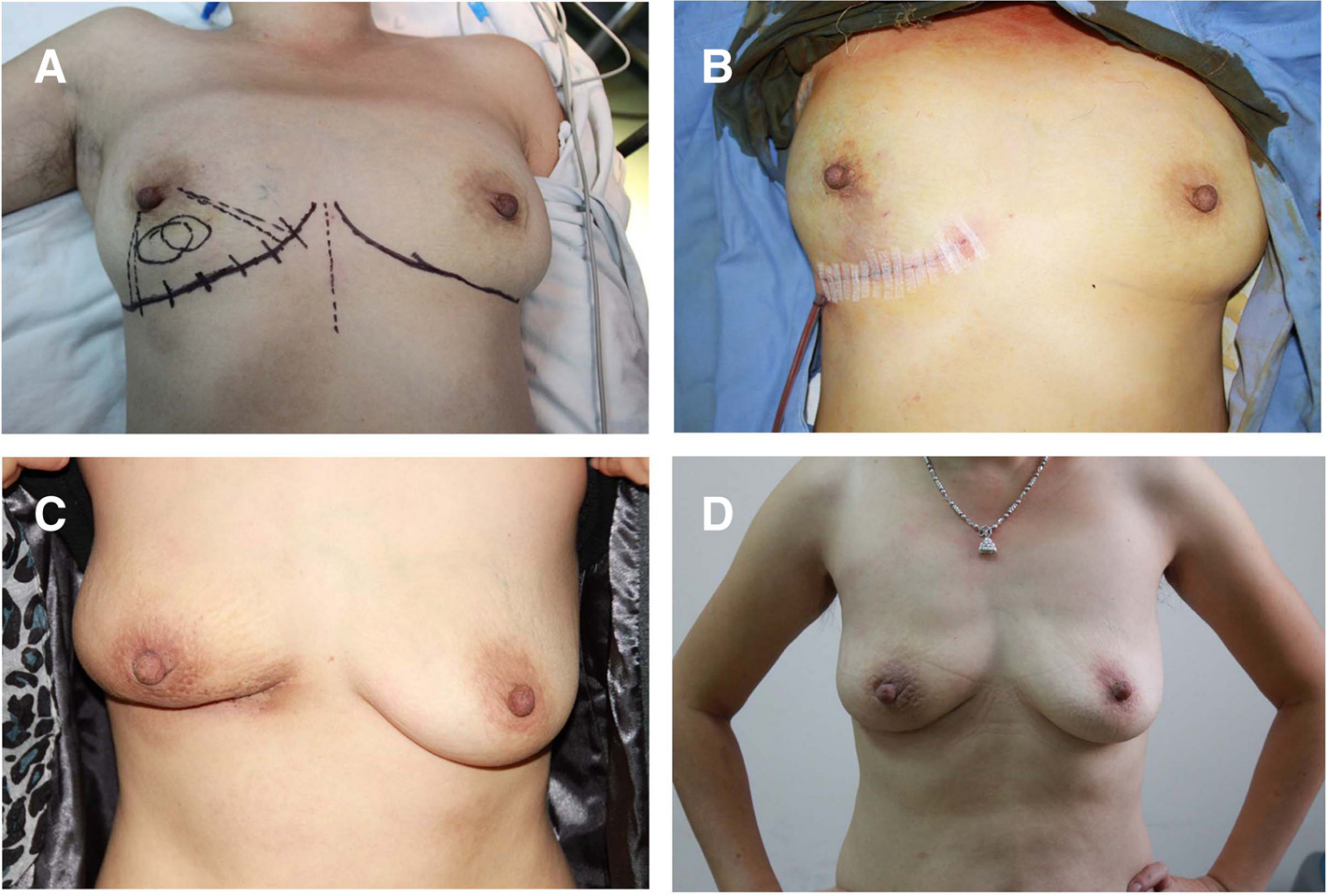
**The omentum seems less susceptible to radiation. (A)** Ductal carcinoma *in situ* in the lower inner quadrant of the right breast. **(B)** Oncoplasty with omentum. The outcome **(C)** 4 months after surgery, 3 months after radiation, asymmetry demonstrated; **(D)** 11 months after surgery, 10 months after radiation, recovered with symmetry.

Whole breast radiation was delivered after surgery. One case of ductal carcinoma *in situ* in the lower inner quadrant of the right breast developed breast asymmetry due to skin contraction 3 months after radiation. However, the cosmetic result was much improved 10 months after radiation without clinical intervention (Figure 3).

Two cases had recurrences. One patient had local recurrence in the reconstructed breast 6 months after surgery. Pathological examination did not show any tumor tissue in the displaced omentum after mastectomy. Three months later, she developed liver and bone metastases during salvage chemotherapy. This patient died 11 months after surgery. Pathological result demonstrated triple-negative breast cancer by IHC stain with intravascular carcinomatous emboli. Ki-67 was 30%, and 7 of 23 axillary lymph nodes were involved. Another recurrent patient was also triple negative. Ki-67 was 5%. Tumor size was 4 × 3 cm with 1 of 12 lymph nodes involved. Two years after the surgery, metastases in the lung, bone, and liver were detected without local recurrence. Further chemotherapy was declined, and close monitoring was still undergoing.

Cosmetic outcomes were assessed by patients and the surgeon independently (Table 5). The overall oncoplastic satisfaction rates by surgeon and patients were 91.7% and 95.8%, respectively. The main concern of unsatisfactory cases was the asymmetry of the breasts.

**Table 5:** **Cosmetic outcomes**

**Assessor**	**Excellent**	**Good**	**Fair**	**Poor**	**Total**	**Satisfaction rate (%)**
Surgeon	19	3	1	1	24	91.7
Patients	20	3	1	0	24	95.8

## Discussion

BCS is currently one of the preferred surgical methods of breast cancer, especially in the early breast cancer. However, BCS is still not the first choice for many Chinese early-breast cancer patients [3]. They tend to have smaller-sized breasts with larger tumors found at diagnosis. Conventional BCS might not keep a well-shaped breast. In such circumstances, the therapeutic option could be volume displacement or volume replacement.

Theoretically, omental flap could be an ideal tissue of breast autologous replacement for Chinese women. It is soft, rich in blood supply, and resistant to infection. Omental flap can also be easily shaped to fill various defects after BCS. Kiricuta [12] first reported the use of an omental flap to reconstruct the breast and thoracic wall in 1963. In 1993, free omental flap harvested laparoscopically was first reported by Saltz [13] to cover a large soft tissue defect. Cothier-Savey [5] and Jimenez [6] reported breast reconstruction with laparoscopically obtained omental flap respectively in the early 21st century. Later, Zaha from Japan reported their experiences on the immediate breast reconstruction with laparascopically harvested omental flap after BCS in 2006 [7] and in 2010 [8,9]. The results showed that it was a safe and feasible technique with less trauma and low incidence of complications. Nevertheless, the development of this technique was stagnant in Western countries, possibly due to the popular prosthesis reconstruction and the volume limitation of omentum. However, the latter might not be a problem for most oriental females with relatively small breasts. Therefore, the oncoplastic breast surgery with omental flap harvested by laparoscopy might be an alternative surgical choice for oriental females.

The advantages of this approach were described as followed:

1. The laparoscopic harvesting was minimal invasive for donor site. Patients felt less postoperative pain and recovered uneventfully. There were no laparoscopic complications such as infection, gastrointestinal perforation, incisional hernia, abdominal hemorrhage, and so on. Only four cases had mild epigastric discomfort and relieved spontaneously.
2. The omental flap was rich in blood supply. Only three cases developed small necrotic nodules without compromising the cosmetics of the reconstructed breast.
3. Compared with the muscle flap, the omental flap was easily shaped for various breast defects. The breast reconstructed by the omentum seemed to be softer, more natural in appearance, and insusceptible to radiation.

The main disadvantage of this procedure was that it was impossible to predict precisely the volume of the omental flap preoperatively. The omentum varied in thickness and surface area, and there was no direct relation between the omentum volume and the patient’s body mass. Even the most advanced radiological technology could not predict the volume precisely. Therefore, it was necessary to assess the volume of the omentum in laparoscopy at the beginning of the surgery. Only if the omentum volume was sufficient to fill the estimated defect, the surgical procedure would proceed according to the plan. We used a laparoscopic bag for sample retrieval of 60, 80, 130, and 200 ml to measure the flap volume for the latest case. The omentum and excised mammary tissue were put into the sample retrieval bag for volume evaluation respectively to make a match. The invention of precise tool for flap volume measurement was essential which was explored by a group of medical engineering staff now.

Secondly, this approach may require a higher cost, longer operative time, assistance of laparoscopy, and additional incision to retrieve the omentum. Besides, difficulty may occur in situations needing a re-excision.

We supposed that this procure was useful for a selected group of women with small breasts to undergo BCS. A further study was required to confirm the oncological safety of this procedure in cases of high-risk recurrence.

## Conclusion

OBS with laparoscopically harvested omental flap might be a feasible technique with a good cosmetic outcome. It may be suitable for a selected group of women with small breasts to undergo BCS. A large-sample study is required to evaluate long-term oncological safety in future.

## Abbreviations

ALND: axillary lymph nodes dissection
BCS: breast conserving surgery
GEAV: gastroepiploic artery and vein
OBS: oncoplastic breast surgery
SLNs: sentinel lymph nodes
SNB: sentinel node biopsy
